# Comparison of Cardiorespiratory Effects of Two Balanced Anesthesia Protocols in Baboons (*Papio hamadryas*) Undergoing Laparoscopic Salpingectomy

**DOI:** 10.3390/vetsci12121134

**Published:** 2025-11-29

**Authors:** Roberta Pizzi, Claudia Piemontese, Caterina Vicenti, Elena Barazia, Marzia Stabile, Claudia Acquafredda, Luca Lacitignola, Marta Guadalupi, Pietro Laricchiuta, Francesco Staffieri

**Affiliations:** 1 Section of Veterinary Clinics and Animal Production, Department of Precision and Regenerative Medicine and Ionian Area (DiMePre-J), University of Bari, 70010 Valenzano, Italy; claudia.piemontese@uniba.it (C.P.); caterina.vicenti@uniba.it (C.V.); marta.guadalupi@uniba.it (M.G.);; 2 Department of Veterinary Medicine, University of Sassari, Via Vienna 2, 07100 Sassari, Italy; e.barazia@studenti.uniss.it; 3 Zoosafari, 72015 Fasano, Italy

**Keywords:** non-human primates, pneumoperitoneum, laparoscopic surgery, salpingectomy, ketamine, medetomidine, cardiorespiratory

## Abstract

Laparoscopic salpingectomy is a minimally invasive surgery that requires careful anesthetic management due to the effects of intra-abdominal gas insufflation. In this retrospective study, baboons *(Papio hamadryas)* were treated with two induction protocols: medetomidine–ketamine (MK; *n* = 16) and medetomidine–tiletamine–zolazepam (MZ; *n* = 12) via intramuscular injection. A laryngeal mask (LMA) was used for airway management and anesthesia was maintained with isoflurane in 100% oxygen. For statistical analysis, the following parameters were analyzed via two-way ANOVA: heart rate (HR), respiratory rate (RR), systolic, diastolic, and mean arterial blood pressure (SAP, DAP, and MAP), end-tidal carbon dioxide (EtCO_2_), and peripheral oxygen saturation (SpO_2_) recorded five minutes before pneumoperitoneum (PREP), after abdominal insufflation (PP1), at 10 (PP2) and 20 (PP3) minutes post-insufflation, and 5 min after pneumoperitoneum interruption (POSTP). HR and RR were statistically significantly higher (*p* < 0.05) in the MK group compared to the MZ group at all time points of the study. EtCO_2_ was significantly higher (*p* < 0.05) in the MZ group at PP2, PP3, and POSTP time points. The incidence of hypotension was significantly greater in the MZ group (45.5%) compared to the MK group (6.25%). Hypercapnia was observed in all baboons sedated with the MZ protocol compared to 12.5% of the MK group. As a result, the MK protocol provided greater cardiorespiratory stability during laparoscopic surgery.

## Simple Summary

This retrospective study aimed to evaluate the cardiorespiratory impact of two different sedation protocols in baboons (*Papio hamadryas*) undergoing laparoscopic surgery. The animals received either a combination of medetomidine and ketamine or medetomidine with tiletamine/zolazepam. Airway management was performed using a laryngeal mask (LMA) and general anesthesia was maintained using isoflurane in 100% oxygen with spontaneous ventilation. An increased degree of cardiorespiratory depression was observed in subjects sedated with the medetomidine–tiletamine–zolazepam protocol. This was characterized by both a lower heart and respiratory rate, higher incidence of hypotension, and hypercapnia. As a result, this protocol was proven to be less suitable for laparoscopic procedures, which inherently impact respiratory and hemodynamic function compared to the medetomidine–ketamine protocol, which provided greater cardiovascular stability and sensitivity to rises in end-tidal carbon dioxide. Furthermore, this study confirmed the safe use of an LMA in this species.

## 1. Introduction

*Papio hamadryas* baboons are the largest non-anthropomorphic monkeys, which belong to the Old World monkey family. Males typically weigh 17–30 kg, while females range from 10 to 17 kg and reach sexual maturity at around four years of age, with interbirth intervals of about two years. The female reproductive cycle averages 30 days, and the ovulation period may be identified by observing tumescence of the genital area [1,2,3].

Baboons raised in captivity require an effective birth control program. Female contraception can be achieved either through pharmacological treatments, which require regular administration, or through salpingectomy, a surgical procedure that demands careful postoperative care [4,5,6]. Laparoscopic salpingectomy offers several advantages, including smaller incisions, reduced postoperative pain, and faster recovery, and it has been shown to be a safe and effective method for baboons [7].

During laparoscopic procedures, increased intra-abdominal pressure causes the compression of abdominal aorta, inferior vena cava, and splanchnic vessels. This results in an increase in systemic vascular resistance, which in an euvolemic patient is observed through a rise in mean arterial pressure (MAP) and a reduction in cardiac output due to a decrease in venous return [8,9,10]. The use of carbon dioxide (CO_2_) to achieve pneumoperitoneum produces an increase in arterial CO_2_ partial pressure (PaCO_2_). Additionally, elevated intra-abdominal pressure displaces the diaphragm cranially, reducing pulmonary compliance. This leads to pulmonary atelectasis and a decrease in tidal volume, which further contributes to higher CO_2_ levels [11,12]. The combination of these mechanisms induces a compensatory increase in respiratory rate [10]. Optimal anesthetic management is essential to minimize physiological impact during laparoscopic surgery; however, very little data has been reported on non-human primates (NHPs), particularly baboons [5,13].

Guidelines from zoo and wildlife organizations recommend several drugs to anesthetize and immobilize baboons [2,14]. Among the main agents available, ketamine and tiletamine, which are non-competitive NMDA receptor antagonists, have been widely investigated in baboons [15,16,17]. Their use is characterized by immobilization, marked muscle rigidity, and cardiovascular effects. Tiletamine has a faster onset of action and longer duration compared to ketamine, and it is commercially available in a combined formulation with zolazepam [18,19]. Other benzodiazepines, such as midazolam, are administered orally in NHPs as anxiolytics prior to anesthesia induction or combined with dissociative agents and opioids via parenteral routes, which may contribute to respiratory depression [20,21,22]. Dissociative agents are often combined with α2-agonists to achieve smooth sedation, optimal muscle relaxation, and enhanced analgesia. Medetomidine is a highly selective α2-agonist (1620:1, α2:α1 selectivity) and its use has been studied in NHPs, including baboons [6,23,24]. Its effects can be reversed by administering atipamezole, an α_2_-antagonist [25,26].

Recently, Scardia et al. found the combination of medetomidine/tiletamine–zolazepam suitable for baboons undergoing laparoscopic salpingectomy [13]. However, no other studies have specifically investigated the impact of different anesthetic drugs during laparoscopic procedures in baboons.

This study aimed to compare two anesthetic protocols employed in a population control program in a baboon troop, involving laparoscopic salpingectomy. We hypothesized that an immobilization protocol based on ketamine would provide a more stable cardiovascular and respiratory profile compared to the use of tiletamine–zolazepam.

## 2. Materials and Methods

This retrospective study collected results based on a clinical observational study approved by the Ethical Committee for Clinical studies by Section of Veterinary Clinics and Animal Production, Department of Precision and Regenerative Medicine and Ionian Area (DiMePre-J) of University of Bari “Aldo Moro” (Approval no: 05/2020).

### 2.1. Animals

Adult and subadult (>2 years) baboons hosted at ZooSafari (Fasano, BA, Italy) underwent elective laparoscopic salpingectomy for a population control program, performed by an experienced veterinary surgeon. Animals received two different anesthetic protocols based on the specific experience of the two veterinarians in charge for the procedures; therefore, allocation to the protocols was not randomized. Three days prior to the scheduled surgery, a harem was separated from the main troop and transferred to a designated area.

### 2.2. Anesthetic Management

Animals were fasted for 15 h, with water available until 8 h before surgery. They were restrained in a crush cage, identified by their microchip numbers, and sedative drugs were administered via intramuscular injection. The two anesthetic protocols employed were as follows: ketamine (Ketavet 100 100 mg/mL, MSD Animal Health S.r.l, Milano, Italia) (3–7 mg/kg) combined with medetomidine (Domitor 1 mg/mL, Zoetis s.r.l., Rome, Italy) (50–100 µg/kg) for the first group (MK group); and tiletamine–zolazepam (Zoletil 100 mg/mL, Virbac S.r.l., Milan, Italy) (2 to 4 mg/kg) combined with medetomidine (20–60 µg/kg) for the second group (MZ group). Drug dosages were calculated based on estimated body weight and adjusted according to the experience of the zoo veterinarian. Accurate body weights were obtained once the animals were deeply sedated. In case of inadequate sedation, a supplemental dose corresponding to half of the initial drug combination was administered.

A 22-gauge venous catheter was placed in the cephalic vein, and following induction a blood sample was collected for complete blood count and biochemical profile analysis.

Animals were positioned in dorsal recumbency on the surgical table and propofol (PropoVet, Zoetis Italia S.r.l., Rome, Italy) was administered to achieve an adequate jaw tone for the placement of a second-generation LMA (LMA Supreme™, The Laryngeal Mask Company Limited, Le Rocher, Victoria, Mahe, Seychelles) appropriately sized for each animal (size 1: <5 kg; size 2: 10–20 kg) [13]. The LMA was inserted with gentle caudal pressure until resistance was felt, after which the cuff was inflated. Proper positioning and seal were verified for each animal by closing the adjustable pressure-limiting valve and squeezing the reservoir bag to achieve an airway pressure of 20 cmH_2_O, checking for any air leakage. Confirmation of adequate positioning was also obtained by observing an adequate capnographic waveform. A maximum of two attempts were allowed for laryngeal mask placement; in case of failure, endotracheal intubation was performed.

Baboons were connected to a rebreathing circuit with spontaneous ventilation. Isoflurane (IsoFlo^®^, Zoetis Italia S.r.l., Roma, Italy) was administered in 100% oxygen via a vaporizer set between 1 and 1.5% and the percentage adjusted according to anesthetic depth. A mainstream capnograph (EMMA^®^ capnograph, Masimo Corporation 52 Discovery, Irvine, CA, USA) was used to measure end-tidal carbon dioxide concentration (EtCO_2_) and respiratory rate (RR). A multiparametric monitor (Compact 5, medical ECONET, Im Erlengrund 20, Oberhausen, Germany) recorded heart rate (HR), systolic (SAP), diastolic (DAP), and mean arterial pressure (MAP) using the oscillometric method, with a cuff positioned on the mid-forearm region. A pulse oximeter was applied to the tongue to assess peripheral oxygen saturation (SpO_2_). The adequacy of the anesthetic plane was monitored by evaluating jaw tone, palpebral reflex, eye position, and hemodynamic parameters. All animals received the following as standard perioperative treatment: Ringer’s solution I.V. (5 mL/kg/h) (Ringer Lattato, Fresenius Kabi, Mirandola, Italy), meloxicam (Metacam, Boehringer Ingelheim, Milan, Italy) at 0.2 mg/kg IV, and cefazoline (Cefazolina Teva, Teva) at 20 mg/kg IV. Surgery was initiated once an adequate surgical plane of anesthesia had been reached. Intra-abdominal pressure was maintained at 6–8 mmHg throughout the procedure. Rescue analgesia was administered as a fentanyl bolus (2 µg/kg IV) (Fentadon, Dechra Veterinary Products, Turin, Italy) in case of a sudden increase (>30%) of RR and/or MAP and/or HR.

Hypotensive episodes, defined as a MAP below 60 mmHg for more than 5 min, were managed by carefully adjusting the depth of anesthesia through a gradual reduction in the isoflurane concentration by 0.5%. If hypotension persisted, a maximum of two fluid boluses (5 mL/kg over 10 min) of Ringer’s lactate solution were administered. If the animal did not respond to adjustments in the depth of anesthesia or to fluid boluses, dopamine was infused at a rate of 5–9 µg/kg/min. Hypercapnia, defined as an EtCO_2_ greater than 55 mmHg, was managed with manual intermittent positive pressure ventilation in the event of a further increase in EtCO_2_ above 65 mmHg and/or respiratory rate (RR) < 5 and/or SpO_2_ < 95%. At the end of the surgery isoflurane was turned off and baboons were placed in lateral recumbency and treated with ivermectin (Ivomec, Boehringer Ingelheim Animal Health Italia S.p.a., Milano, Italia) (0.2 mg/kg SC) and amoxicillin (Betamox LA, Vétoquinol Italia S.r.l., Bertinoro, Italia) (25 mg/kg IM).

The laryngeal cuff was deflated and removed when the palpebral reflex reappeared. The animals were transferred to a warm recovery area and monitored up to full recovery, which was when the animal had regained a standing position.

### 2.3. Statistics

All physiological parameters (HR, SAP, MAP, DAP, EtCO_2_, RR, and SpO_2_) were recorded at specific time points: five minutes before pneumoperitoneum (PREP), immediately after abdomen insufflation (PP1), at 10 min (PP2) and 20 min (PP3) from the start of surgery, and 5 min after pneumoperitoneum was stopped (POSTP). All recorded parameters were statistically analyzed using MedCalc 12.7.0.0. software. Normal distribution of data collected during each study time was confirmed with the Shapiro–Wilk test. Median, mean value, and confidence interval (95% CI) were calculated for all data. Physiological parameters were compared between the two groups over different time intervals via a repeated-measures two-way ANOVA test. Post hoc analysis was performed using Tukey’s test. *p* < 0.05 was considered statistically significant.

## 3. Results

Twenty-eight baboons, weighing between 4 and 15 kg, were considered for the purposes of this study. The MZ protocol was applied in 12 animals, while in the remaining 16 cases the MK protocol was used. All animals completed the study uneventfully. The mean body weight was 9.6 kg (95% CI: 7.3–11.9) in the MZ group and 10.7 kg (95% CI: 9.7–11.6) in the MK group. The MK group included 5 subadult and 11 adult baboons, while the MZ group included 3 subadult and 9 adults.

In the MZ group, the effective dosage of tiletamine–zolazepam ranged from 2.3 to 10.4 mg/kg (mean: 4.4 mg/kg, 95% CI: 2.8–6.1), while for medetomidine it ranged from 10 to 40 µg /kg (mean: 20 µg /kg, 95% CI: 17–30). In the MK group, ketamine was administered at doses ranging from 3 to 7 mg/kg (mean: 4.37 mg/kg, 95% CI: 3.6–5.15), and medetomidine from 50 to 100 µg/kg (mean: 80 µg /kg, 95% CI: 63–90). In the MZ group, propofol was administered at doses ranging from 2 to 3.5 mg/kg (mean: 2.68 mg/kg; 95% CI: 2.2–3.1), whereas in the MK group it was at doses ranging from 2.5 to 4.6 mg/kg (mean: 3.42 mg/kg; 95% CI: 2.6–4.4). No animal required an additional dose of sedative. The mean surgical time was 36.5 min in the MZ group (95% CI: 30.5–42.5) and 27 min in the MK group (95% CI: 24–30).

**Figure 1:**
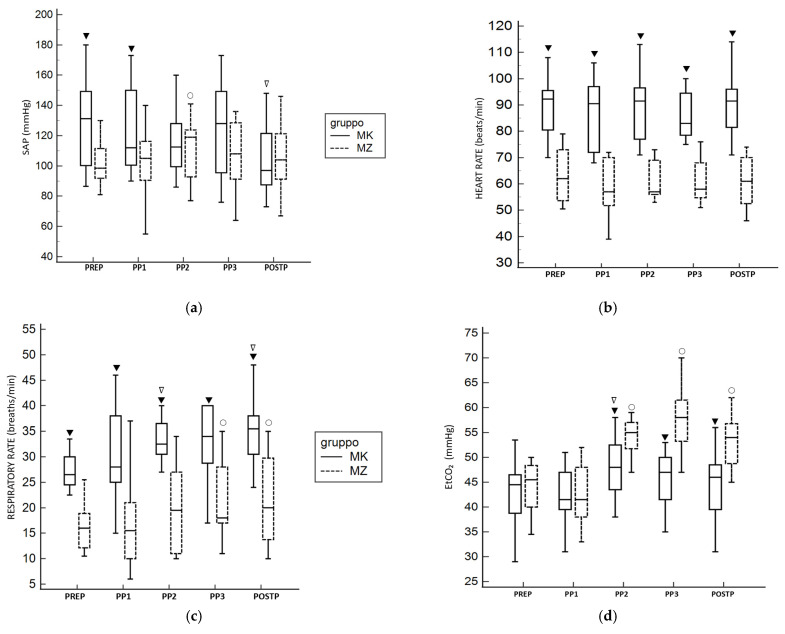
The figures show cardiorespiratory parameters across groups at different time points during the study. Systolic arterial pressure (**a**) resulted statistically significantly higher in the MK group for PREP and PP1 time points. Heart rate (**b**) and respiratory rate (**c**) resulted significantly higher in the MZ group at all time points. EtCO_2_ (**d**) resulted statistically significantly higher in the MZ group at PP2, PP3, and POSTP. The line within the box represents the median value. The upper and lower limits of the boxes represent the 25th and 75th percentiles of the data, respectively. The whiskers extend from the minimum to the maximum value. ▼ = *p* < 0.05 for MK compared to MZ; ○ = *p* < 0.05 compared to MZ PREP; ∇ = *p* < 0.05 compared to MK PREP.

The mean values and CI 95% for HR, MAP, RR, EtCO_2_, and SpO_2_ for both groups are presented in Table 1. A statistically significant difference was observed between the two groups for SAP. The MK group showed higher values compared to the MZ group at PREP and PP1. Within the same group, compared to PREP, the MZ protocol exhibited higher values of SAP at PP2 while the MK group showed lower values at POSTP (Figure 1a). Regarding HR, the MK group showed higher values compared to the MZ group at all time points during the study (Figure 1b).

For RR, the MK group showed significantly higher values during the entire study time compared to the MZ group. Within the same group, compared to PREP, the MK group exhibited higher RR values at PP2 and POSTP, while the MZ group showed higher values at PP3 and POSTP (Figure 1c). EtCO_2_ levels were significantly higher in the MZ group at PP2, PP3, and POSTP compared to PREP; conversely, in the MK group a significantly higher value was observed at PP2 compared to PREP. A statistical difference was observed between two groups, with a higher value of EtCO_2_ at PP2, PP3, and POSTP for the MZ group (Figure 1d). For all other parameters analyzed, no statistically significant differences were reported between the two groups. None of the animals required rescue analgesia during the surgical procedure.

**Figure 2:**
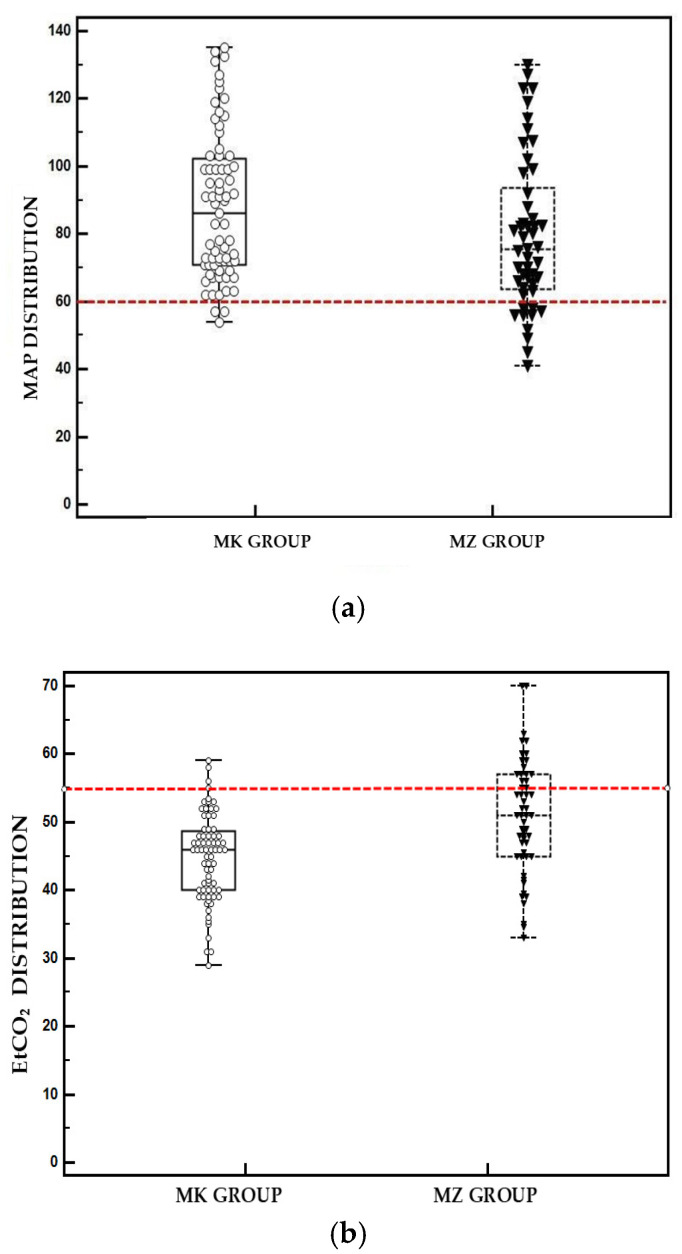
Box plots describing the distribution of (**a**) hypotension and (**b**) hypercapnia between groups. The dashed line represents the threshold values for hypotension (60 mmHg) and hypercapnia (55 mmHg).

In the MZ group, 5 out of 12 baboons (41.6%) experienced transient hypotension episodes during pneumoperitoneum, which were resolved by adjusting the depth of the anesthetic plan and administering fluid boluses. The incidence of hypotension was significantly lower in the MK group, with only 1 episode recorded among 16 animals (6.25%). At least one episode of hypercapnia was recorded in all cases of the MZ group, while in the MK group the incidence was significantly lower (*p* < 0.001), with 2 of 16 animals (12.5%) affected. However, no animal required manual intermittent positive pressure ventilation. Figure 2 shows box plots representing the distribution of hypercapnia and hypotension episodes in the animals throughout the different time points of the study. Recovery was uneventful for all animals and within one hour of the end of the surgery.

## 4. Discussion

Considering the physiological alterations associated with laparoscopic surgery, the use of the medetomidine–ketamine (MK) protocol resulted in a lower impact on both respiratory and cardiovascular parameters compared to the medetomidine–tiletamine–zolazepam (MZ) protocol during laparoscopic salpingectomy in baboons.

Despite the theoretical increase in MAP during pneumoperitoneum and the sympathomimetic action of dissociative agents, the MZ group showed a higher incidence of hypotension and a significantly lower SAP during PREP and PP1 time points. Additionally, heart rate was also significantly lower in the MZ group compared to the MK group. This finding may be attributed to the typical behavior of α2-agonist on the heart rate, an effect that appears to be more pronounced in combination with tiletamine–zolazepam [27]. These observations align with previous studies reporting decreased cardiocirculatory performance in NHPs anesthetized with tiletamine–zolazepam [28,29].

In this specific study, the absence of mechanical ventilation may have highlighted the different impact of the two protocols on respiratory drive.

During pneumoperitoneum, the physiological response to hypercapnia typically involves an increase in respiratory rate to facilitate CO_2_ elimination [10]. This compensatory mechanism was not evident in the MZ group, as shown in Figure 1c, indicating the presence of respiratory depression. Consequently, a progressive increase in EtCO_2_ was observed throughout the surgical procedure. Although there was hypoventilation in the MZ group, no hypoxemia (SpO_2_ < 94%) was recorded at any time point. However, this result was achieved through the administration of pure oxygen, which may have altered the representation of peripheral oxygen saturation [15,30,31].

Conversely, in the MK group, EtCO_2_ levels were better regulated during pneumoperitoneum. It seems that the combination of MK showed less interference with the physiological compensatory respiratory drive response to CO_2_ insufflation than MZ. In Figure 1d, an increase in ETCO_2_ was observed after 10 min of pneumoperitoneum, followed by a decrease during the subsequent phase. This reflects the respiratory center sensitivity to rising CO_2_ levels. In fact, an increase in respiratory rate was observed, resulting in a corresponding decrease in EtCO_2_ (Figure 1c,d). In this group, mild hypercapnia was observed in only two subjects.

Based on these data, the MZ protocol was associated with greater cardiovascular and respiratory depression, resulting in more pronounced physiological alterations compared to the MK group. This negative impact could be attributed to the presence of zolazepam. This observation is consistent with previous data on the depressive effects of benzodiazepines in NHPs and other species [19,32,33]. While benzodiazepines are considered drugs with a good safety margin when administered alone, their concomitant use with other agents, such as α2-agonists, may enhance negative cardiorespiratory effects [34]. This association may potentiate the effects on the central nervous system, leading to increased muscle relaxation and deeper levels of sedation [29,35].

Nevertheless, it was interesting to observe that all animals tolerated pneumoperitoneum well, particularly in the MK group, where an adequate respiratory drive was maintained. This outcome was further facilitated by careful surgical technique, which ensured intra-abdominal pressures did not exceed 8 mmHg. These findings suggest that laparoscopic salpingectomy is well tolerated in these animals, even with spontaneous ventilation. The use of an appropriate sedation protocol can further guarantee this outcome.

Regarding the use of an LMA, no animals experienced complications during its application. This study confirms previously reported findings in the literature regarding its safety and efficacy in NHPs [13,36,37]. The baboon’s trachea is shorter compared to other primates, which can complicate intubation. The use of an LMA overcomes this anatomical limitation, facilitating airway management and preventing potential airway damage and infection [15,38]. The main risk associated with LMA application is regurgitation, particularly during laparoscopic procedures which may increase intra-abdominal pressure. This can result in aspiration pneumonia due to the airways remaining patent and unprotected [39]. However, this risk can be reduced through an adequate fasting period and by using second-generation LMA, which offers improved sealing and enhanced safety. No episodes of regurgitation occurred in this study.

The study presented several limitations. Primarily, its retrospective design may have influenced data collected, as there was no randomization of animals between the two groups. The limited sample size may have reduced the statistical power for some variables that did not reach significance, although significant results were obtained for others. Drug dosages were not standardized, which may have contributed to variability in cardiovascular and respiratory parameters among individuals within the same group. Furthermore, due to logistics of the zoo, complete and adequate monitoring of recovery quality was not possible. A recent study by Amari et al. evaluated three different sedation protocols in baboons, reporting that animals treated with the combination containing tiletamine–zolazepam had lower recovery scores compared to those sedated with ketamine-based protocols [27].

## 5. Conclusions

Data collected in this study proved that the combination of medetomidine and ketamine resulted in less cardiovascular and respiratory depression compared to a combination of medetomidine with tiletamine–zolazepam in baboons undergoing laparoscopic salpingectomy. Moreover, the study confirms that LMAs represent a valid and safe alternative to orotracheal intubation in these animals. Further studies are warranted to investigate the optimal dosage of medetomidine–ketamine combination and its effects on the quality of recovery.

## Figures and Tables

**Table 1:** Mean value and 95% CI of heart rate (HR), mean arterial pressure (MAP), diastolic arterial pressure (DAP), systolic arterial pressure (SAP), end-tidal carbon dioxide (EtCO_2_), respiratory rate (RR), and peripheral oxygen saturation (SpO_2_). ▼ = *p* < 0.05 for MK compared to MZ; ○ = *p* < 0.05 compared to MZ PREP; ∇ = *p* < 0.05 compared to MK PRE.

Parameters	Group	PREP	PP1	PP2	PP3	POSTP
**HR** **(beats/min)**	MZ	59 (45–72)	58 (50–66)	61 (56–67)	61 (56–67)	51 (55–68)
	MK	88 (83–94) ▼	87 (80–94) ▼	88 (82–95) ▼	86 (77–95) ▼	90 (84–95) ▼
**MAP** **(mmHg)**	MZ	74 (52–96)	73 (59–88)	85 (64–105)	77 (57–96)	82 (65–99)
	MK	93 (82–105)	92 (81–104)	88 (77–100)	89 (66–113)	78 (68–89)
**DAP** **(mmHg)**	MZ	62 (40–83)	61 (45–78)	66 (44–88)	63 (44–81)	66 (49–83)
	MK	77 (66–88)	78 (67–88)	75 (64–85)	73 (53–93)	65 (55–76)
**SAP** **(mmHg)**	MZ	92 (67–118)	104 (88–119)	110 (92–127) ○	106 (89–124)	107 (91–123)
	MK	128 (109–141) ▼	123 (108–137) ▼	119 (104–134)	123 (91–155)	104 (92–116) ∇
**EtCO_2_ (mmHg)**	MZ	41 (32–49)	42 (38–47)	55 (51–60) ○	58 (53–62) ○	53 (49–57) ○
	MK	43 (40–46)	43 (40–47)	48 (45–51) ▼∇	46 (40–51) ▼	45 (41–48) ▼
**RR (breaths/min)**	MZ	16 (11–21)	18 (11–26)	20 (14–26)	21 (15–27) ○	21 (16–27) ○
	MK	28 (24–32) ▼	31 (26–36) ▼	33 (28–37) ▼∇	33 (25–41) ▼	34 (31–38) ▼∇
**SpO_2_ (%)**	MZ	97 (95–98)	97 (96–99)	98 (97–99)	98 (96–99)	98 (98–99)
	MK	98 (98–99)	98 (97–99)	97 (96–98)	97 (94–99)	97 (95–98)

## Data Availability

The original contributions presented in this study are included in the article. Further inquiries can be directed to the corresponding author.

## Abbreviations

The following abbreviations are used in this manuscript:

ANOVA	Analysis of variance
CI	Interval confidence
CO_2_	Carbon dioxide
DAP	Diastolic arterial pressure
EtCO_2_	End-tidal carbon dioxide
HR	Heart rate
IM	Intramuscular
IV	Intravenous
LMA	Laryngeal Mask airway
MAP	Mean arterial pressure
MK	Medetomidine–ketamine group
MZ	Medetomidine–tiletamine–zolazepam group
NHPs	Non-human primates
PaCO_2_	Partial pressure of arterial carbon dioxide
POSTP	5 min after pneumoperitoneum interruption
PP1	Immediately after starting pneumoperitoneum
PP2	Pneumoperitoneum 10 min
PP3	Pneumoperitoneum 20 min
PREP	Pre-pneumoperitoneum
RR	Respiratory rate
SAP	Systolic arterial pressure
SpO_2_	Peripheral oxygen saturation
